# Synergistic Effect of CNT and N-Doped Graphene Foam on Improving the Corrosion Resistance of Zn Reinforced Epoxy Composite Coatings

**DOI:** 10.3390/polym16243513

**Published:** 2024-12-17

**Authors:** Yana Mao, Shufu Liu, Shizhong Liu, Guodong Wu, Qi Liu, Xusheng Du

**Affiliations:** 1 School of Civil Engineering, Lanzhou Jiaotong University, Lanzhou 730070, China; 0116016@stu.lzjtu.edu.cn (Y.M.); liusz@mail.lzjtu.cn (S.L.); 2 Gansu Province Highway Aviation Tourism Investment Group Co., Ltd., Lanzhou 730030, China; 3 China Communications Construction Road & Bridge Southern Engineering Co., Ltd., Beijing 101100, China; 447094717@163.com (G.W.); 15561429472@163.com (Q.L.); 4 Institute of Advanced Wear & Corrosion Resistant and Functional Materials, Jinan University, Guangzhou 510632, China

**Keywords:** epoxy composite coating, corrosion, three-dimensional graphene, carbon nanotube, hybrid fillers, self-repairing

## Abstract

The synergistic effect of CNT and three-dimensional N-doped graphene foam (3DNG) on improving corrosion resistance of zinc-reinforced epoxy (ZRE) composite coatings was studied in this work. Although CNT itself was demonstrated to be effective to promote the anti-corrosion performance of the ZRE coating, the incorporation of additional 3DNG leads to further enhancement of its corrosion resistance under the synergistic effect of the hybrid carbon nanofillers with different dimensions. Both the content of the carbonaceous fillers and the ratio between them affected the performance of the coating. The optimal content of hybrid filler in the coating was determined to be only 0.1% with 3DNG:CNT = 1:3. With the modification of hybrid fillers, the corrosion current of the coating could be reduced by more than six orders of magnitude. Additionally, the immersion test of the pre-scratched coating directly demonstrated the evident contribution of the hybrid fillers to the sacrificial anode-based surface protection mechanism of the coating. These results confirmed the synergistic effect of the hybrid 1D and 3D carbonaceous fillers on promoting the corrosion inhibition of their coating, which could be promising for application in other functional composites.

## 1. Introduction

Zinc-rich epoxy (ZRE) composites have been widely used as the anti-corrosive coating for metallic substrates [1]. Besides the common barrier protection mechanism of the composite primers, the cathodic protection effect of zinc powders in the composite coating also contributes significantly to the corrosion resistance of the coating. Due to its excellent chemical stability, high conductivity, environmental friendliness and high surface area, carbon nanomaterials have been extensively used as functional fillers in the polymer composite coatings [2], including those filled with and without zinc powders.

There are many types of carbon nanomaterials, including both 1D, 2D and 3D ones. The 1D CNTs have been added into ZRE coatings to improve their anti-corrosion properties [3,4]. It was reported that, with the incorporation of 2 wt% CNTs, the hybrid epoxy coating has the highest anti-corrosion performance [4]. Different from 1D CNT, 2D graphene (G) has more advantages, as it is capable of providing an additional barrier to the coating due to its own excellent impermeability to corrosives [2,5,6,7]. Therefore, graphene and its derivatives, including graphene oxide and reduced graphene oxide (rGO), have been utilized to incorporate into ZRE coatings [8,9,10,11,12,13,14,15,16,17,18]. In a recent work, GO was in situ reduced and modified with the curing agent of epoxy during the fabrication of ZRE paint, and at an optimal content of 0.3 wt%, the corrosion current of the resulting coating was reduced to 1.69 × 10^−12^ A·cm^−2^, which is five orders of magnitude lower than that of neat ZRE, demonstrating the great positive effect of graphene on the cathodic protection performance of the coating [9]. Moreover, in order to utilize the 3D structure of the unique carbon fillers, ZRE coatings modified with 3D graphene and N-doped reduced graphene oxide (3DNG) were developed very recently [19]. Thanks to their spacial effect on reduction in graphene agglomeration, the construction of a conduction network in the composite [20,21], their ZRE coating was promoted evidently at a very low filler content, which is superior to that modified with the conventional 2D graphene sheets.

The hybrid fillers with different dimensions have been demonstrated to have synergistic effects on various properties of their composites, including the mechanical [22], electrical [23] and thermal conduction and wear resistance [24]. The synergistic effect of 0D carbon dots and 2D BN nanosheets on improving the corrosion resistance of their epoxy coating was demonstrated [25]. Both 0D silicone and nanodiamond have been used to modify 2D graphene oxide for fabrication of epoxy vinyl ester composite coatings as well, and their excellent long-term anti-corrosive performance was confirmed [26]; the impedance value |Z|_0.01Hz_ of the coating was ~2–3 orders larger than that of the pure polymer coating after soaking for 120 days in a salt solution. On the basis of previous reports, it can be speculated that one can utilize the hybrid filler of 3D G and 1D CNT to promote the cathodic protection of the ZRE coating. Unfortunately, little research on this aspect has been reported so far.

In this work, the effect of the hybrid filler of 3DNG and CNT on improving the corrosion resistance of zinc-rich epoxy coatings will be studied. Based on the previous work on the 3D graphene filler-modified ZRE coating, CNT will be additionally incorporated to promote the cathodic protection function of the ZRE coating to the metallic substrate, and the synergistic effect of these two nanocarbon fillers with different dimensions will be investigated. The content of the hybrid filler will be optimized to maximize this effect. Moreover, the anti-corrosion behavior of the coating will be studied with various methods, including the long-term immersion test and the element mapping of the scratch-defected ZRE coating samples after immersion.

## 2. Materials and Methods

### 2.1. Preparation of the Carbon Nanomaterial-Modified ZRE Coatings

The 3DNG was prepared by the flame combustion of GO coating polyurethane foam according to the method reported previously [20]. A certain amount of 3DNG or CNT (length of 0.5–2 μm) was added into the paint diluent, followed by the addition of epoxy resin E44 and zinc micro-flakes (lateral size: 5~10 μm). The mixture was stirred vigorously for 2 h. Polyamide 651 was then added into the mixture (its ratio to epoxy resin = 1:2) and continuously stirred for 10 min to obtain the carbon nanomaterial-modified ZRE paints. Zinc flakes and epoxy diluent accounted for 35% and 20% of the total paint, respectively. The fabrication process of the ZRE paints modified by different contents of hybrid fillers (3DNG:CNT = 1:3) was consistent with that for ZRE modified with a single filler. According to the type and content of carbon nanofillers, i.e., 0.2 wt% CNT, 0.2 wt% 3DNG, 0.2 wt% (3DNG + CNT), 0.1 wt% (3DNG + CNT) and 0.05 wt% (3DNG + CNT), the resulting ZRE paints were labeled as ‘02_CNT ZRE’, “02_3DNG ZRE’, ‘02_3DNG-CNT ZRE’, ‘01_3DNG-CNT ZRE’ and ‘005_3DNG-CNT ZRE’, respectively. Neat ZRE without adding any carbon nanofiller was also prepared as the reference sample. Rectangular Q235 steel plates were used as metal substrates, which were ultrasonically cleaned with acetone after sandblasting. The paints were applied onto the metal surface with an applicator and cured at 60 °C for 24 h.

### 2.2. Characterization Methods

A typical three-electrode system was assembled with a saturated calomel electrode (SCE) as the reference electrode, platinum sheet as the counter electrode and the coated Q235 plates as the working electrode, whose exposed area in the corrosive solution was 1 cm^2^. A 3.5 wt% NaCl solution was used as the corrosive solution for the corrosion tests. The electrochemical impedance spectroscopy (EIS) and potentiodynamic polarization (or so-called Tafel) curves of the ZRE coatings were measured on a CHI760E electrochemical analyzer (Chenhua CHI Co., Shanghai, China). The EIS plots of the ZRE coatings under different immersion times were carried out in the frequency range of 10^5^~10^−2^ Hz, with a sinusoidal voltage disturbance amplitude of 5 mV. ZView software was used to fit the EIS data. The Tafel tests were also performed before and after 300 h soaking in the salt solution. For the immersion test of the scratch-defected coating sample, the scratch was made by a blade to expose the Q235 steel substrate. The morphology of the sample was observed on a scanning electron microscope (SEM; ULTRA55, Zeiss, Germany).

## 3. Results and Discussion

### 3.1. Anti-Corrosion Performance of Hybrid Carbon-Modified ZRE Coatings

**Figure 1:**
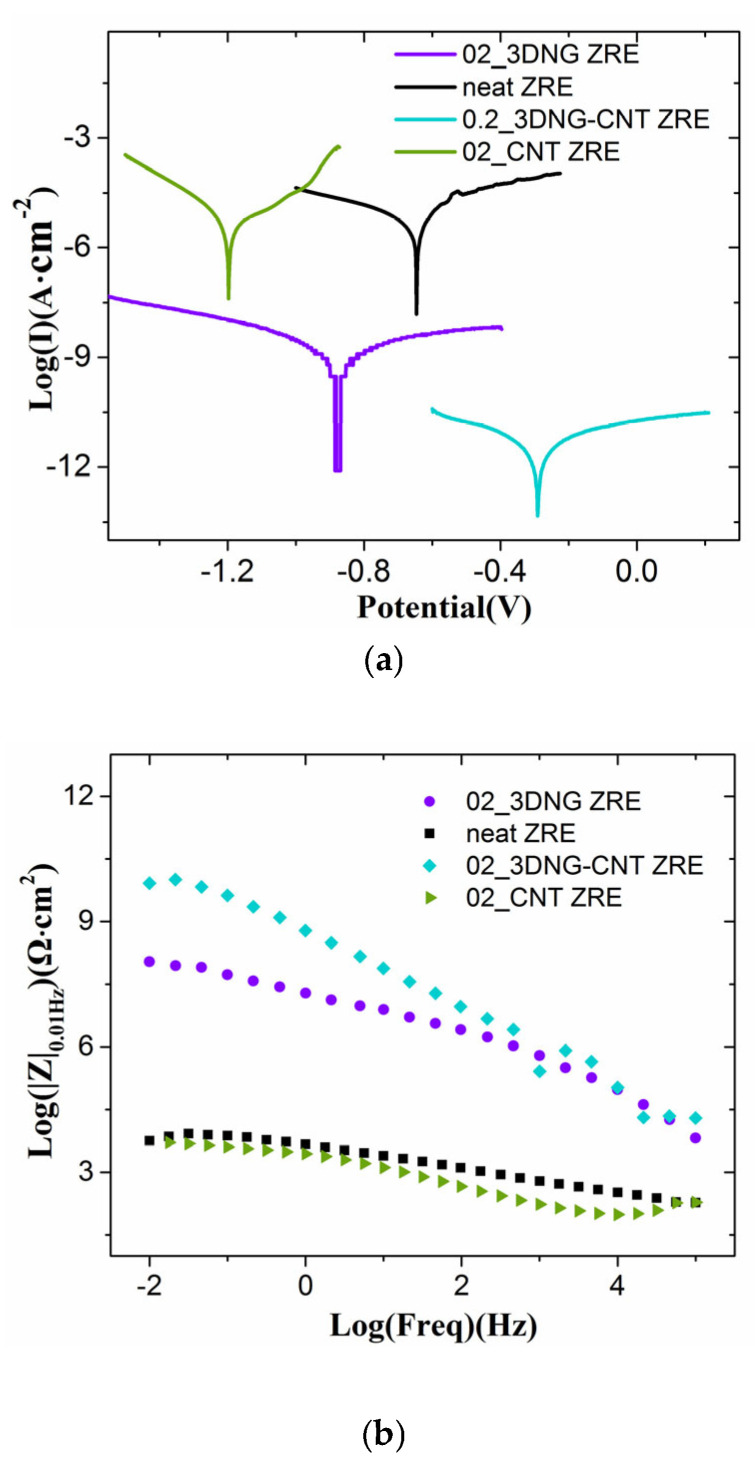
(**a**) Tafel curves and (**b**) Bode plots of neat ZRE and hybrid filler-modified ZRE coatings.

In consideration of the practical application of ZRE paints, it is highly demanding for reducing the content of expensive graphene and its derivatives in the ZRE premiers. According to the recent work on 3DNG-modified ZRE coatings, its optimal content was determined to be 0.05 wt% [19]. On the base of this work, a small amount of commercial CNTs (0.15 wt%) was further added into the paint to observe if there was any hybrid effect between these two carbon nanomaterials with different dimensions. Obviously, the content of the total hybrid carbon nanofiller in the ZRE paint would be 0.2 wt%. The Tafel curve of the resulting 02_3DNG-CNT ZRE is shown in Figure 1a, and its corrosion current density (Icorr) and polarization resistance (R_L_) were calculated to be 3.07 × 10^−12^ A·cm^−2^ and 1.39 × 10^11^ Ω·cm^2^, respectively (Table 1). Compared to the ZRE modified with 0.05 wt% 3DNG, i.e., 9.56 × 10^−12^ A·cm^−2^ for Icorr and 4.55 × 10^11^ Ω·cm^2^ for R_L_ [19], the decreased Icorr and increased R_L_ of 02_3DNG-CNT ZRE confirmed that it promoted corrosion resistance. Besides the corrosion current intensity and polarization resistance, the low frequency impedance modulus (|Z|_0.01Hz_) obtained from the EIS data was another important parameter, which is widely used to evaluate the surface protection of a coating. Evidently, the larger |Z|_0.01Hz_ shown in the Bode plot of the 02_3DNG-CNT ZRE coating in Figure 1b was also consistent with the Tafel analysis results. In fact, CNTs had been demonstrated to be effective nanofillers in reducing the porosity of the coating, hindering the diffusion of corrosion species, thus improving the anti-corrosion property of their coatings [4]. Moreover, 1D CNTs could work well with 3D graphene to form the conductive network to activate more zinc particles in the epoxy matrix to exert their cathodic protection function.

As the synergistic effect of fillers with different dimensions on various performance of the polymer composites had been demonstrated previously [22,23,24,25,26], one was expected to see whether such an effect contributes to the improvement of the corrosion resistance of ZRE coatings. ZRE coatings incorporated with the same content of a single carbon filler were fabricated, and their corrosion resistances were characterized. As shown in Figure 1 and Table 1, compared with that of 02_3DNG-CNT ZRE, both Tafel curves of 02_3DNG ZRE and 02_CNT ZRE moved upwards, and their Icorr values were evidently larger than that of 02_3DNG-CNT ZRE, especially for that of 02_CNT ZRE. The Icorr value of 02_CNT ZRE was even the same as that of neat ZRE, indicating the neglect contribution of CNTs to the cathodic protection of the ZRE coating. Such an effect of CNTs on ZRE was similar to the report by [27], where single-walled CNTs were utilized. Remarkably, it could be found that a reduction of more than six orders of magnitude in Icorr was realized, thanks to the utilization of hybrid fillers in the ZRE coating, as well as an increase of approximately eight orders of magnitude in RL, as shown in Table 1. The Bode plot shown in Figure 1b also indicates that the 02_3DNG-CNT ZRE had a much larger |Z|_0.01Hz_ than both 02_3DNG ZRE and 02_CNT ZRE. All these results demonstrate the synergistic effect of 3DNG and CNTs on enhancing the corrosion protection of ZRE coatings.

**Figure 2:**
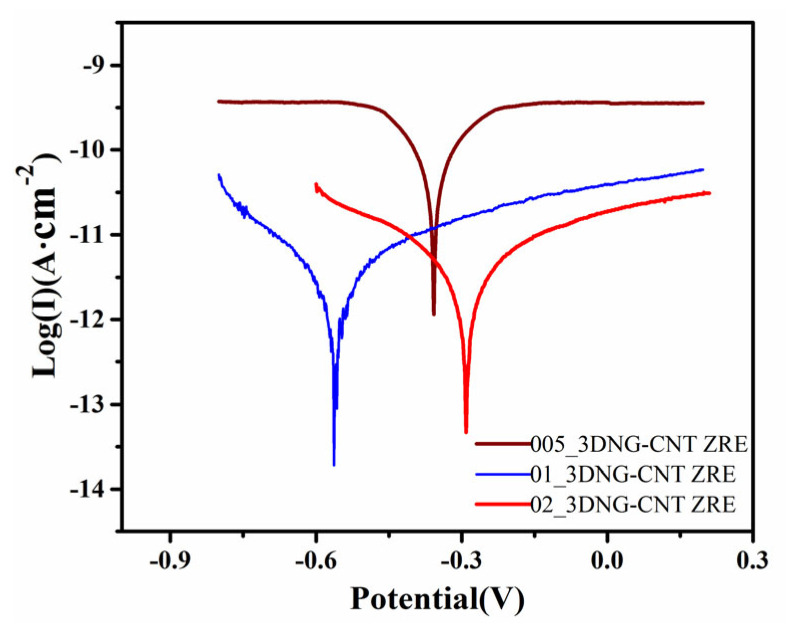
Tafel curves of nanocarbon-modified ZRE-coated Q235 samples.

Based on the aforementioned results, the effects of different hybrid filler contents on the corrosion inhibition performance of ZRE coatings were studied. Interestingly, as shown in Figure 2, the Icorr of the coating was further reduced to 2.84 × 10^−12^ A·cm^−2^ when the content of the hybrid filler was reduced by half to 0.1 wt%, and its R_L_ increased accordingly as well (Table 2), indicating its improved anti-corrosion performance at an even lower content of hybrid filler. However, with a further decrease of the hybrid filler content to 0.05 wt%, its Icorr was greatly increased to 1.08 × 10^−10^ A·cm^−2^, which was 38-fold that of 01_3DNG-CNT ZRE (Table 2). This meant that the effect of the hybrid filler on improving the anti-corrosion performance of 3DNG-CNT ZRE depended on its content, and the optimal content of the hybrid filler in this work was 0.1 wt%, which was comprised of only 0.025 wt% 3DNG and 0.075 wt% CNT.

### 3.2. Long-Term Immersion Test of Hybrid Filler-Modified Coatings

**Figure 3:**
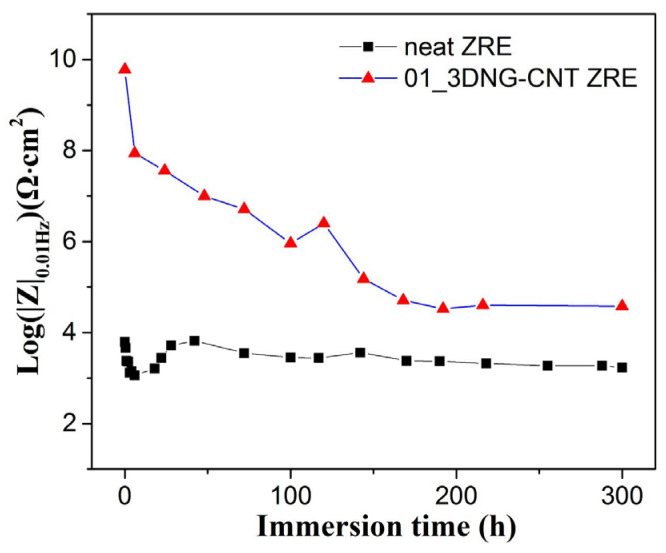
The dependence of |Z|_0.01Hz_ of neat ZRE and 01_3DNG-CNT ZRE coatings on the immersion time.

The |Z|_0.01Hz_ values of the 01_3DNG-CNT ZRE coating were monitored for up to 300 h to study its long-term anti-corrosion behavior. As shown in Figure 3, the |Z|_0.01Hz_ ~ immersion time curves of the hybrid filler-modified coating dropped with the immersion time initially. It reached a relatively stable state later during the entire 300 h immersion test. This might be caused by the corrosion products in situ generated after the rapid corrosion of zinc particles, which hindered the further corrosion of the steel substrate. Moreover, it could be observed that its |Z|_0.01Hz_ values were always larger than those of neat ZRE, indicating it promoted long-term corrosion inhibition performance.

**Figure 4:**
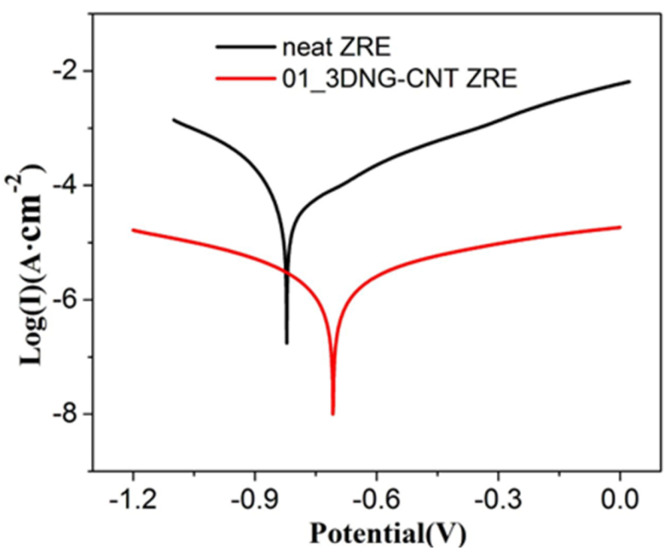
Tafel curves of neat ZRE and 01_3DNG-CNT ZRE coatings after 300 h immersion in 3.5 wt% NaCl solution.

The potentiodynamic polarization test of the hybrid filler-modified ZRE-coated Q235 samples was also performed to characterize its long-term corrosion resistance behavior. Figure 4 shows the Tafel curves of the 01_3DNG-CNT ZRE coating after 300 h immersion in a 3.5 wt% NaCl solution, and the corresponding corrosion parameters are presented in Table 3. Evidently, 01_3DNG-CNT ZRE displayed a much smaller Icorr value (6.94 × 10^−7^ A·cm^−2^) compared to that of neat ZRE (4.87 × 10^−5^ A·cm^−2^). Meanwhile, its R_L_ was still much larger than that of neat ZRE. This confirmed its superior long-term anti-corrosion performance as well.

**Figure 5:**
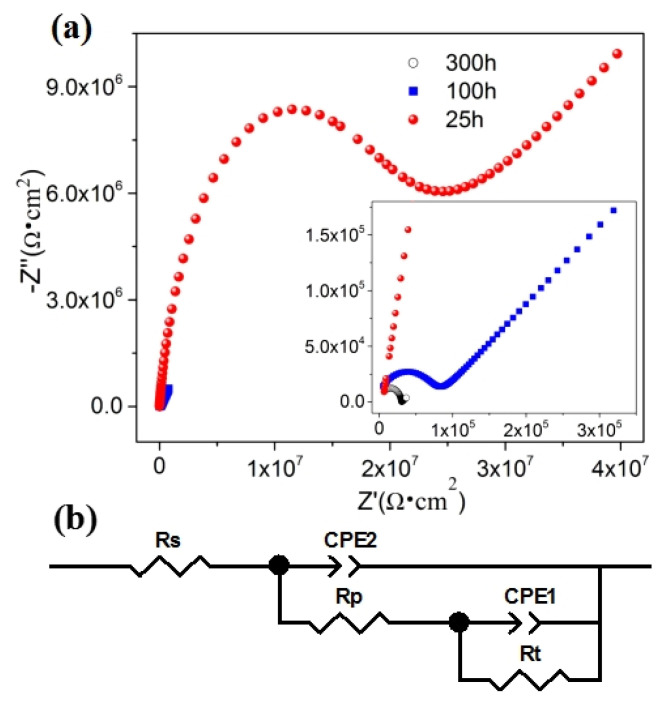
(**a**) Nyquist plots of 01_3DNG-CNT ZRE coating on Q235 sample immersed in NaCl solution at 25 h, 100 h and 300 h and (**b**) schematic diagrams of equivalent analog circuits for EIS analysis of the hybrid nanocarbon-modified ZRE.

The Nyquist plots of the hybrid filler-modified ZRE coatings soaked in the corrosive solution at three time stages (25 h, 100 h and 300 h) are shown in Figure 5a. The schematic diagram of the equivalent analog circuit for fitting the EIS data is shown in Figure 5b. In the circuits, Rt relates to the electrochemical corrosion of zinc particles during the cathodic protection process, while the pore resistance (Rp) of the coating and the CPE-2 capacitance relate to the entire coating’s impermeability [16]. The detailed as-fitted data for the samples are shown in Table 4. It can be observed that the Rt values of the 01_3DNG-CNT ZRE decreased with the extension of immersion, implying the deterioration of the corrosion resistance of the coating. Meanwhile, the CPE-2 values of the ZRE coating increased with the immersion time. It had been suggested that the water absorption behavior of the polymer coating could be evaluated with the CPE-2 value [9,28]. The increased CPE-2 values meant deteriorated impermeability of the 01_3DNG-CNT ZRE coating to water, which coincided with the increased Rp values as well (Table 4).

### 3.3. The Cathodic Protection Behavior of the Hybrid Filler-Modified Coating

**Figure 6:**
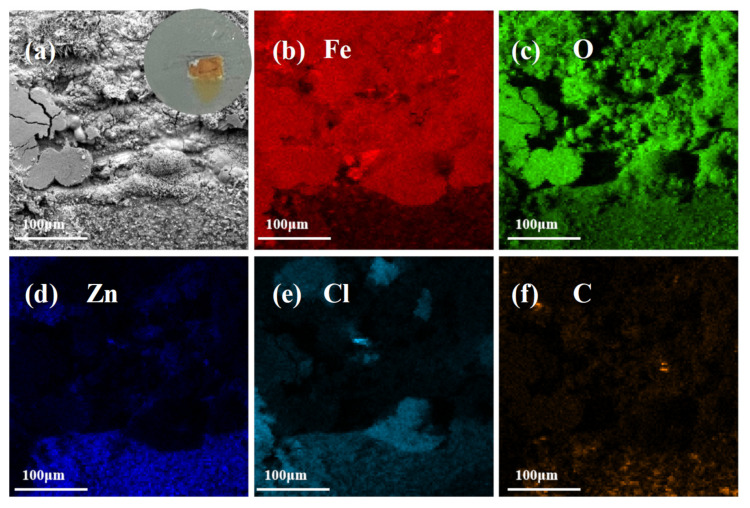
Images of scratched area of neat ZRE soaked in 3.5 wt% NaCl solution for 12 h: (**a**) SEM image and inset digital photo; the element mapping of (**b**) Fe, (**c**) O, (**d**) Zn, (**e**) Cl and (**f**) C.

**Figure 7:**
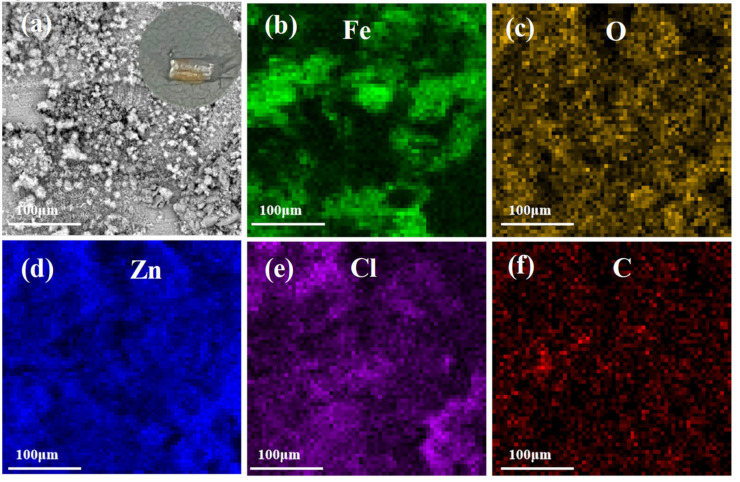
Images of scratched area of the hybrid filler-modified ZRE-coated steel sample soaked in 3.5 wt% NaCl solution for 12 h: (**a**) SEM image and inset digital photo and the element mapping of (**b**) Fe, (**c**) O, (**d**) Zn, (**e**) Cl and (**f**) C.

In order to explore the cathodic protection behavior of the ZRE coating, the Q235 samples coated with either neat ZRE or 01_3DNG-CNT ZRE were deliberately scratched deeply enough to expose the steel substrate and immersed in a 3.5 wt% NaCl solution to detect their corrosion resistance mechanism. The inset images in Figure 6a and Figure 7a show the digital photos of the exposed steel surface of neat ZRE and 01_3DNG-CNT ZRE-coated Q235 plates, respectively, after 12 h immersion in the corrosive solution. Obviously, after soaking for 12 h, the scratch area in the neat ZRE became totally brown, indicating the occurrence of severe corrosion in the steel substrate. In contrast, such a severe corrosion phenomenon was absent from the scratched 01_3DNG-CNTZRE sample (inset image in Figure 7a) soaked for the same time, and only a small area displayed a very light brown color. This directly demonstrated the better cathodic protection function of the 01_3DNG-CNT ZRE coating for the steel substrate.

The SEM and elemental mapping analyses of the corroded scratch regions of both neat ZRE and the hybrid filler-modified ZRE coating were further carried out. As shown in Figure 6a, the major area of the corroded scratch of the sample was covered by the corrosion products of the steel substrate, for instance, products α-Fe_2_(OH)_3_Cl, Fe_3_O_4_ and α-Fe_2_O_3_ [26], except the one-third area at the bottom part, which was the unscratched part of the neat ZRE coating. The EDS mapping images of Figure 6b–d confirm this and demonstrate the severely corroded substrate of the neat ZRE-coated sample at the corrosive conditions. Different from the Fe-based major corrosion product of the steel substrate in the scratched neat ZRE-coated sample, the Zn element dominated in the element mapping image of the hybrid filler-modified ZRE-coated sample and was distributed relatively uniformly in the studied area, as shown in Figure 7d. This implied that the corrosion product generated from the Zn powders in the coating could form a continuous layer, which isolated the steel substrate from the corrosive solution and thus improved the corrosion resistance of the sample. The contents of the Cl element (Figure 7e) and C element (Figure 7f) obtained in the scratch area of the coating should majorly originate from the corrosion products of the Zn powders, such as Hydrozincite Zn_5_(CO_3_)_2_(OH)_6_ and Willemite (Zn_5_(OH)_8_Cl_2_·H_2_O) [19,29,30]. These test results demonstrate the self-repairing characteristic of the 01_3DNG-CNTZRE coating, whose mechanism is similar to those coatings based on the release of organic corrosion inhibitor molecules from the composite coating and isolation of steel substrate from further corrosion by the in situ formed solid Fe^3+^-based complex compounds [31,32,33]. However, to the best of our knowledge, most of the research available so far on the epoxy coating incorporated with the corrosion inhibitors is not a zinc-rich epoxy composite coating. Due to the lack of the essential function of cathodic protection with the sacrificial anode provided by the large amount of Zn particles, they found little utilization as heavy-duty coatings.

**Figure 8:**
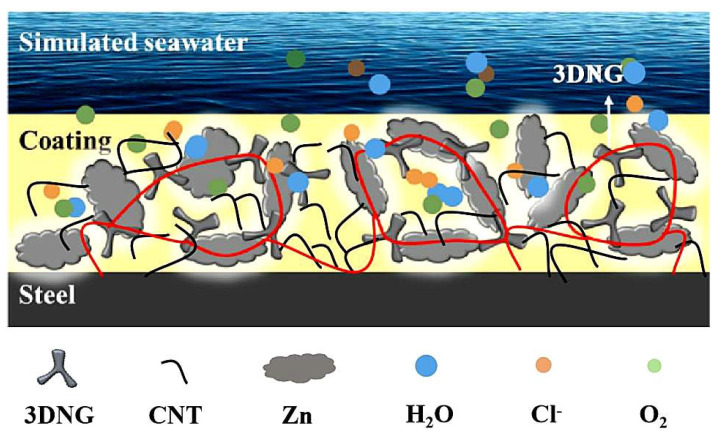
Schematic diagram of the anti-corrosive mechanism of 3DNG-CNT ZRE coating on steel in simulated seawater.

Figure 8 illustrates the anti-corrosion mechanism of ZRE coatings modified with hybrid nanocarbon fillers. The incorporation of 3DNG into the ZRE coating helps to form electro-conductive paths between Zn particles and the steel substrate, as more conduction paths formed between the Zn particles and the steel substrate are conducive to activating more zinc particles to exert their cathodic protection function through the sacrificial-anode electrochemical corrosion reaction. In addition to this, the excellent impermeability of 3DNG to the corrosives also benefits the barrier property of its ZRE coating. However, taking into account the large size of 3DNG (micron scale), there are still some minor spaces to be filled to build a more effective conductive network. With further incorporation of CNTs, micropores in the coating can be eliminated more or less with the nanofillers, therefore hindering the corrosive invasion through the coating. Moreover, due to the electrical connection provided by the 1D CNTs, more Zn particles will be linked into the conductive network made of 3DNG, CNT, Zn particles and the steel substrate for exerting their cathodic protection function (see the red line in Figure 8), thereby maximizing the sacrificial-anode roles of zinc in the entire coating system.

## 4. Conclusions

The effect of the hybrid filler of 3DNG and CNT on improving the cathodic protection function of a ZRE coating was exploited for the first time. Compared with neat ZRE and 0.2 wt% CNT-incorporated ZRE, the Icorr of ZRE modified with 0.2 wt% hybrid filler (0.05 wt% 3DNG and 0.15 wt% CNT) was reduced by more than six orders of magnitude. Moreover, it was ~1/32 that of ZRE modified with the same content of 3DNG. Therefore, the synergistic effects of these two nanocarbon fillers with 1D and 3D dimensions on the cathodic protection of the ZRE coating were confirmed. The dependence of the hybrid filler-modified coating on its content was also revealed, and the optimal content of the hybrid filler was 0.1 wt%, which was comprised of only 0.025 wt% 3DNG and 0.075 wt% CNT. The long-term cathodic protection behavior of the 3DNG–CNT-modified ZRE coating was studied, and the corresponding corrosion mechanism was proposed. Additionally, the immersion test of the scratch-defected coating demonstrated the self-repairing capability of the hybrid filler-modified ZRE coating, which resulted from its promoted sacrificial-anode-based steel protection function. This work provides a versatile methodology to improve the cathodic protection function of a zinc-containing composite coating by utilizing the hybrid nanocarbon fillers with different dimensions, which might be applied to other composite coatings.

## Figures and Tables

**Table 1:** Icorr, Ecorr and RL of neat ZRE and nanocarbon-modified ZRE coating samples.

Samples	I_corr_ (A·cm^−2^)	E_corr_ (V)	R_L_ (Ω·cm^2^)
neat ZRE	6.11 × 10^−6^	−0.646	5.67×10^3^
02_3DNG-CNT ZRE	3.07 × 10^−12^	−0.290	1.39×10^11^
02_3DNG ZRE	9.63 × 10^−10^	−0.885	5.09×10^7^
02_CNT ZRE	6.11 × 10^−6^	−1.196	6.47×10^3^

**Table 2:** Icorr, Ecorr and RL of the nanocarbon hybrid filler-modified ZRE coatings.

Samples	I_corr_ (A·cm^−2^)	E_corr_ (V)	R_L_ (Ω·cm^2^)
005_3DNG-CNT ZRE	1.08 × 10^−10^	−0.357	4.03 × 10^9^
01_3DNG-CNT ZRE	2.84 × 10^−12^	−0.561	1.47 × 10^11^
02_3DNG-CNT ZRE	3.07 × 10^−12^	−0.290	1.39 × 10^11^

**Table 3:** Icorr, Ecorr and RL of neat ZRE and 01_3DNG-CNT ZRE-coated Q235 samples after 300 h immersion in 3.5 wt% NaCl solution.

Samples After 300 h Immersion	I_corr_ (A·cm^−2^)	E_corr_ (V)	R_L_ (Ω·cm^2^)
neat ZRE	4.87 × 10^−5^	−0.821	7.76 × 10^2^
01_3DNG-CNT ZRE	1.11 × 10^−6^	−0.707	4.01 × 10^4^

**Table 4:** Equivalent circuit fitting of 01_3DNG-CNT ZRE-coated Q235 sample immersed in 3.5 wt% NaCl solution.

Time (h)	CPE-1 (S·cm^−2^·s^n^)	R_p_ (Ω·cm^2^)	CPE-2 (S·cm^−2^·s^n^)	R_t_ (Ω·cm^2^)
25	1.01 × 10^−9^	1.39 × 10^7^	5.95 × 10^−8^	1.45 × 10^15^
100	3.28 × 10^−9^	8.11 × 10^4^	3.46 × 10^−6^	3.70 × 10^14^
300	5.30 × 10^−10^	2.94 × 10^4^	3.33 × 10^−4^	7.56 × 10^8^

## Data Availability

The original contributions presented in this study are included in the article; further inquiries can be directed to the corresponding authors.
